# A Novel Function of Noc2 in Agonist-Induced Intracellular Ca^2+^ Increase during Zymogen-Granule Exocytosis in Pancreatic Acinar Cells

**DOI:** 10.1371/journal.pone.0037048

**Published:** 2012-05-17

**Authors:** Sho Ogata, Takashi Miki, Susumu Seino, Seiichi Tamai, Haruo Kasai, Tomomi Nemoto

**Affiliations:** 1 Department of Cell Physiology, National Institute for Physiological Sciences, and Graduate University of Advanced Studies (SOKENDAI), Okazaki, Aichi, Japan; 2 Department of Laboratory Medicine, National Defense Medical College Hospital, Tokorozawa, Saitama, Japan; 3 Department of Pathology and Laboratory Medicine, National Defense Medical College, Tokorozawa, Saitama, Japan; 4 Division of Cellular and Molecular Medicine, Kobe University Graduate School of Medicine, Kobe, Hyogo, Japan; 5 Department of Medical Physiology, Chiba University, Graduate School of Medicine, Chiba, Chiba, Japan; 6 Center for Disease Biology and Integrative Medicine, Faculty of Medicine, University of Tokyo, Bunkyo, Tokyo, Japan; 7 Laboratory of Molecular and Cellular Biophysics, Research Institute for Electronic Science, Hokkaido University and Core Research for Evolutional Science and Technology (CREST), Japan Science and Technology Agency (JST), Sapporo, Hokkaido, Japan; Indiana University School of Medicine, United States of America

## Abstract

Noc2, a putative Rab effector, contributes to secretory-granule exocytosis in neuroendocrine and exocrine cells. Here, using two-photon excitation live-cell imaging, we investigated its role in Ca^2+^-dependent zymogen granule (ZG) exocytosis in pancreatic acinar cells from wild-type (WT) and Noc2-knockout (KO) mice. Imaging of a KO acinar cell revealed an expanded granular area, indicating ZG accumulation. In our spatiotemporal analysis of the ZG exocytosis induced by agonist (cholecystokinin or acetylcholine) stimulation, the location and rate of progress of ZG exocytosis did not differ significantly between the two strains. ZG exocytosis from KO acinar cells was seldom observed at physiological concentrations of agonists, but was normal (vs. WT) at high concentrations. Flash photolysis of a caged calcium compound confirmed the integrity of the fusion step of ZG exocytosis in KO acinar cells. The decreased ZG exocytosis present at physiological concentrations of agonists raised the possibility of impaired elicitation of calcium spikes. When calcium spikes were evoked in KO acinar cells by a high agonist concentration: (a) they always started at the apical portion and traveled to the basal portion, and (b) calcium oscillations over the 10 µM level were observed, as in WT acinar cells. At physiological concentrations of agonists, however, sufficient calcium spikes were not observed, suggesting an impaired [Ca^2+^]_i_-increase mechanism in KO acinar cells. We propose that in pancreatic acinar cells, Noc2 is not indispensable for the membrane fusion of ZG *per se*, but instead performs a novel function favoring agonist-induced physiological [Ca^2+^]_i_ increases.

## Introduction

Rab proteins are small G-proteins that coordinate membrane transport in both exocytotic and endocytotic pathways [1]–[5]. They include the subfamilies Rab3 and Rab27, which have been implicated in Ca^2+^-dependent exocytosis [2], [6]–[10]. Noc2 was identified first in endocrine cells, and it was postulated to be a Rab3 effector mediating regulated exocytosis [11], [12]. In fact, overexpression of Noc2 has been reported to enhance the Ca^2+^-dependent exocytosis of large dense-core vesicles from an adrenal pheochromocytoma cell-line, PC12 cells [11]. Experiments on Noc2-deficient (Noc2^−/−^; KO) mice showed that Noc2 is essential for the regulation of secretion in pancreatic endocrine cells [13]. Those experiments suggested that Noc2 positively regulates insulin secretion from pancreatic ß-cells by inhibiting the signaling cascade of a heterotrimeric G protein, Gi/o, and that an interaction between Noc2 and Rab3 or Rab27 is required for this effect. Further, in several exocrine glands (viz., salivary glands, pancreatic exocrine glands, gastric chief cells, the Paneth cells of the jejunum, and Brunner's glands of the duodenum) in KO mice, Noc2 deletion led to accumulations of secretory granules and also to impaired agonist-induced amylase secretion from pancreatic acini [13]. However, it remains unclear how Noc2 deletion might impair zymogen granule (ZG) exocytosis in pancreatic exocrine glands, particularly agonist-induced regulated exocytosis.

Pancreatic acinar cells are considered to be representative cells exhibiting Ca^2+^-dependent ZG exocytosis. In these cells, agonist-induced regulated exocytosis is triggered by increases in the intracellular free Ca^2+^ concentration ([Ca^2+^]_i_), and these increases result predominantly from Ca^2+^ release from the endoplasmic reticulum (ER) via the binding of inositol trisphosphate (IP_3_) to IP_3_-receptor channels (IP_3_-induced Ca^2+^ release; IICR) [14]–[17]. If we are to elucidate the mechanism responsible for impairments of Ca^2+^-dependent ZG exocytosis, it is important to make quantitative analyses of the spatiotemporal changes in [Ca^2+^]_i_ and the number of ZG undergoing exocytosis, as well as an examination of the mode of such exocytosis. However, in exocrine glands this has been difficult to achieve by classical confocal microscopy because of the lack of sufficient tissue depth penetration to allow visualization of the fine organization [18]. Two-photon microscopy has been shown to have the ability both to penetrate deeply into tissues and to excite a number of fluorescent dyes simultaneously [18]–[20]. Taking advantage of these attributes of two-photon microscopy, we previously analyzed spatiotemporal changes in [Ca^2+^]_i_ and sequential compound exocytosis by simultaneous imaging of [Ca^2+^]_i_ and individual exocytotic events in intact acinar cells from exocrine glands [18], [21], [22]. Here, we employed two-photon microscopy for a simultaneous demonstration of increases in [Ca^2+^]_i_ and individual exocytotic events in living pancreatic acini isolated from Noc2-KO mice. Such quantitative imaging suggested that in the exocrine pancreas, Noc2 may not be essential as a Rab effector in ZG exocytosis, but instead may act, upstream of ZG exocytosis, to promote agonist-induced increases in [Ca^2+^]_i_.

## Methods

### Preparation of pancreatic acinar cells from KO and WT mice

This experimental study was carried out in accordance with the recommendations in the *Guide for the Care and Use of Laboratory Animals* of the National Institutes of Health. The protocol was approved by the Committee on the Ethics of Animal Experiments in the National Institute of Physiological Sciences (No. A17-87-107). The generation and characterization of Noc2-KO mice have been described elsewhere [13]. The absence of Noc2 expression in the KO mice was confirmed by Northern blotting, RT-PCR, and Western blotting (data not shown). We found no apparent abnormalities in either the development or behavior of these mice [13]. The preparation of clusters of acini was performed as previously described [17], [20]. Briefly, clusters of acini were isolated from 4- to 7-week-old mice by brief (4 min) digestion with collagenase (1 mg ml^−1^; Wako, Osaka, Japan) followed by gentle trituration. The acini were dispersed in a small chamber and superfused (1 ml min^−1^) with a solution termed Solution-A [150 mM NaCl, 5 mM KCl, 2 mM CaCl_2_, 1 mM MgCl_2_, 10 mM HEPES-NaOH (pH 7.3), and 10 mM glucose] [18], [21]. All chemical substances, except where otherwise stated, were purchased from Nacalai Tesque Co. (Kyoto, Japan).

### Cross-sectional images of intact acini by two-photon microscopy

Isolated clusters of acini were loaded either with the Ca^2+^ indicator fura-2 (K_Ca_: 0.2 µM) or with its low-affinity version fura-2 FF (K_Ca_: 40 µM) [18]. This was achieved by incubation in Solution-A containing the appropriate cell-permeable acetoxymethyl (AM) form [fura-2-AM (10 µM; Molecular Probes, Eugene, OR) or fura-2 FF-AM (20 µM; Tef Lab, Dallas, TX)] for 30 min at room temperature. The acini in the recording chamber were continuously superfused with Solution-A. The [Ca^2+^]_i_ values were calculated from the fluorescence of fura-2 or fura-2 FF, as previously described [18], [23], [24]. [Ca^2+^]_i_ was calculated from the ratio of fura-2 or fura-2 FF fluorescence during stimulation (F) to that obtained before stimulation (F_0_) according to the equation shown below.
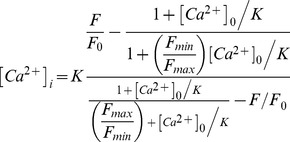
In the equation, [Ca^2+^]_0_ is assumed to be 0.1 µM, and the affinities of Ca^2+^ for fura-2 and fura-2 FF, K, are assumed to be 0.2 and 40 µM, respectively. F_max_ and F_min_ represent fluorescence values for the Ca^2+^-free and Ca^2+^-bound forms of the indicators, respectively, and F_min_/F_max_ values *in vivo* were estimated to be 0.29 for fura-2 and 0.15 for fura-2FF.

The superfusion medium was changed to Solution-A containing a fluorescent fluid-phase polar tracer, sulforhodamine B (SRB; Molecular Probes; 0.5 mM) before the observation period. Individual events of ZG exocytosis were visualized, by means of the extracellular tracer SRB, as the formation of an Ω-shaped profile. That is, after a ZG had fused with the plasma membrane, SRB diffused rapidly from the luminal area through fusion-pores into the individual ZG, leading to the formation of an Ω-shaped profile [25]. The agonists used here were dissolved in this SRB-containing Solution-A, and applied to cells through a glass pipette.

Two-photon excitation imaging of pancreatic acinar cells was performed as described previously [18]. In brief, cells were imaged using an inverted microscope (IX70; Olympus, Tokyo, Japan) equipped with a water-immersion objective lens (UPlanApo60× W/IR; numerical aperture, 1.2). A mode-locked Ti: Sapphire laser (Tsunami; Spectra Physics, Mountain View, CA) was attached to the laser port of a laser-scanning microscope (FV300; Olympus). The laser power at the specimen was 3–5 mW, and the excitation wavelength was 830 nm.

For simultaneous imaging of [Ca^2+^]_i_ and ZG exocytosis, fluorescence (both from fura-2 or fura-2 FF, and from SRB) was measured at 400–550 nm and 570–650 nm, respectively. Fluorescence images were acquired every 0.5 to 2 s. The 12-bit images were analyzed and color-coded using “fall” and “gray” look-up tables, the image-acquisition and analysis software employed being either the Fluoview software of the FV300 microscope or IPLab Spectrum (Scanalytics, Fairfax, VA). Rapid artificial increases in [Ca^2+^]_i_ were triggered by ultraviolet flash photolysis of NP-EGTA, which was preloaded by incubation of acini with 10 µM NP-EGTA-AM (Molecular Probes) in Solution-A for 30 min, as described previously [21].

We carried out a two-tailed Student t-test to assess whether the difference between the means of two groups of measurements was significant. Such a difference was regarded as significant at a probability *p*<0.05.

## Results

### Intracellular localization of zymogen granules

**Figure 1:**
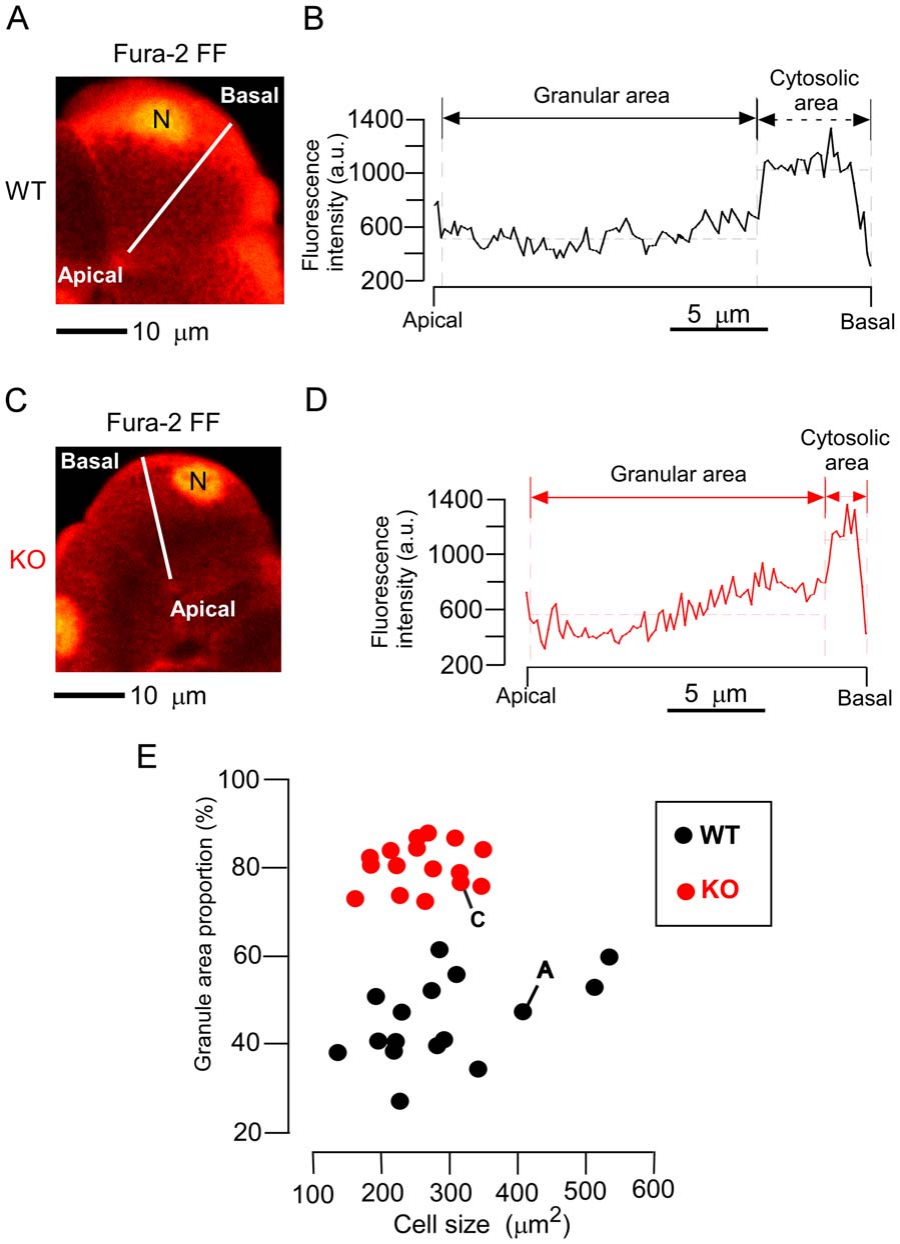
Relative extent of granular area within pancreatic acinar cells of wild-type and knockout mice. *A, C*. Cross-sectional fluorescence images of wild-type (WT; A) and knockout (KO; C) mouse acinar cells loaded with fura-2 FF-AM. N indicates nucleus. *B, D*. Fura-2 FF fluorescence intensity of WT (B) and KO (D) acinar cells, as measured along the solid white lines on the images A and C, respectively. *E*. Plot of cell-size against “granular area proportion” in WT (black dots) and KO (red dots) acinar cells. “Granular area proportion” was calculated by expressing the extent of the low-intensity area as a percentage of the cell area (namely, the sum of low-intensity and high-intensity areas) on the basis of the above findings derived from cross-sectional images. Dots labeled A and C correspond to the cells in images A and C, respectively.

First we examined, using two-photon excitation imaging, the intracellular localization of ZGs within the fura-2 FF-loaded pancreatic acinar cells of KO and WT mice. Cross-sectional fura-2 FF fluorescence images of unstimulated cells displayed a low-intensity area in the apical region and a high-intensity area in the basal region (Fig. 1A, C). Line-mode intensity analysis along the lines shown from the apical to the basal region revealed that in both KO and WT acinar cells, the mean fluorescence-intensity profile was under 700 arbitrary units in the low-intensity area, but above 1,000 in the high-intensity area (Fig. 1B, D). A previous study using multiphoton-excitation images revealed that the low-intensity area represented the accumulated ZGs (namely, the granular area), while the high-intensity area represented the cytosolic area [18]. When the extent of the low-intensity area was expressed as a percentage of that of the cell area (viz., sum of low-intensity and high-intensity areas) on the basis of the above findings in cross-sectional images, this value (the “granular area proportion”) was significantly larger in KO acinar cells than in WT acinar cells [Fig. 1E, *p*<0.05; KO acinar cells: 80.4±1.3% (mean ± SE), 72.3–87.6%, n = 16; WT acinar cells: 45.4±2.5%, 27.0–61.3%, n = 16]. When a cone-shaped model was adopted as a simple representation of the three-dimensional shape of acinar cells, the mean volume occupied by the granular area was estimated to be 72.1% in KO acinar cells against only 30.6% in WT acinar cells (volumes being in linear units to the power three, while areas are linear units to the power two). These findings suggested that ZGs accumulated more abundantly in KO than in WT acinar cells, as previously described [13].

### Spatiotemporal analysis of zymogen granule exocytosis induced by agonist stimulation

**Figure 2:**
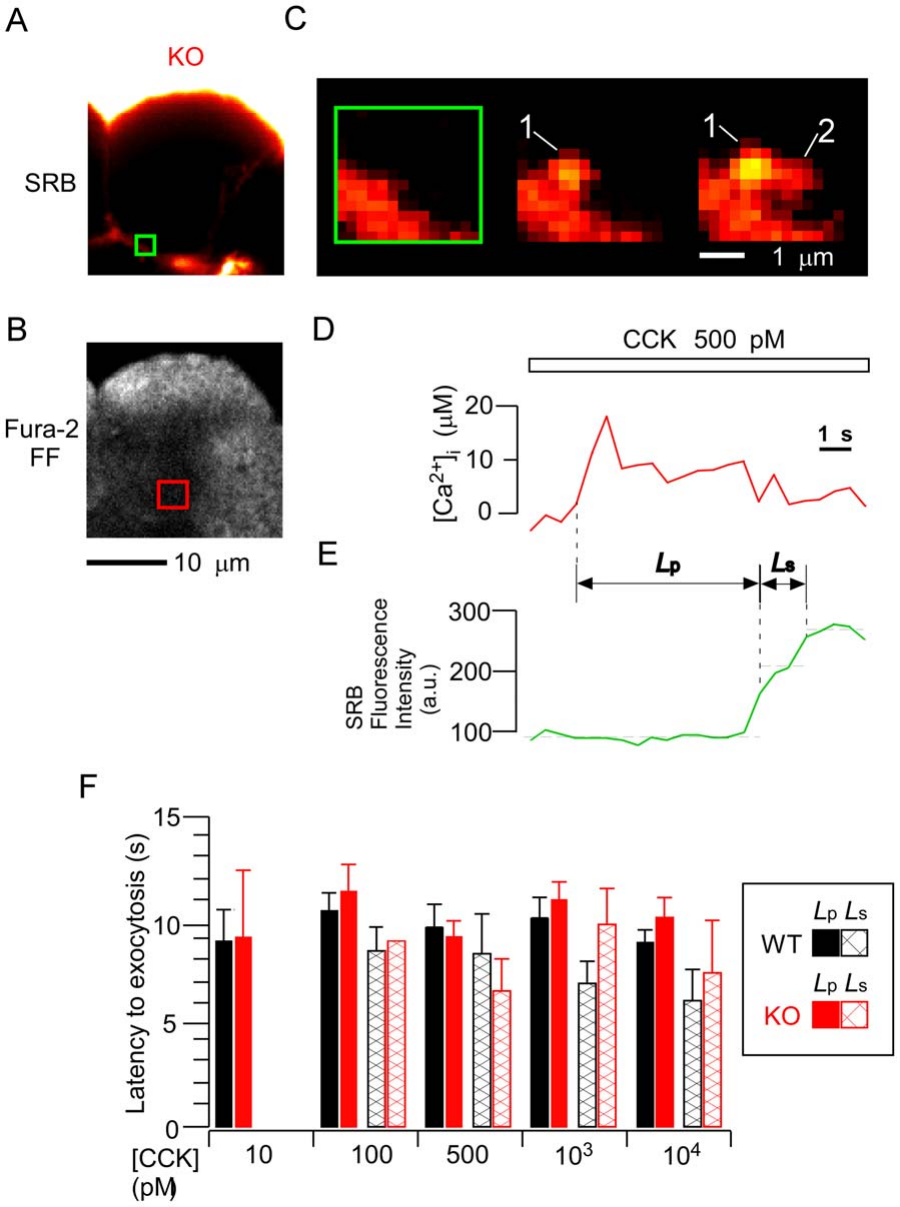
Mode of exocytotic events and their latencies in knockout acinar cells on cholecystokinin stimulation. *A, B.* Simultaneous cross-sectional SRB (A) and Fura-2 FF (B) fluorescence images of knockout (KO) acinar cells. *C.* Magnifications of SRB fluorescence images in the apical region (green box in A) during application of 500 pM cholecystokinin (CCK). The Ω-shaped profiles (1, 2) represent zymogen granules that fused sequentially at 6 and 7.5 seconds after calcium spike. *D, E*. Time course plots of changes in [Ca^2+^]_i_ (D) in the apical region (red box in B) and of changes in SRB fluorescence intensity (E) in the apical region (green box in A). Vertical broken lines correspond to the times at which the 3 images shown in C were obtained. *Lp* indicates latency to primary exocytotic event from calcium spike, and *Ls* latency to next fusion from pre-fused exocytotic event. *F.* Dependency on the concentration of CCK shown by *Lp* (solid bars) and *Ls* (cross-hatched bars) in WT (black) and KO (red) acinar cells. *Ls* data are not shown for 10 pM CCK because secondary exocytotic events were not observed. Data are means ± SE of values from the following number of Ω-shaped profiles: ***Lp***, 16 (10 pM), 121 (100 pM), 86 (500 pM), 62 (1 nM), or 55 (10 nM) in WT cells, and 4 (10 pM), 34 (100 pM), 78 (500 pM), 64 (1 nM), or 32 (10 nM) in KO cells; ***Ls***, 45 (100 pM), 21 (500 pM), 25 (1 nM), or 14 (10 nM) in WT cells, and 1 (100 pM), 7 (500 pM), 19 (1 nM), or 9 (10 nM) in KO cells.

To find the explanation for the impaired mechanism of amylase secretion in KO acinar cells that was observed biochemically in a previous study [13], we first examined a typical ZG-exocytosis response (latency between a calcium spike and ZG exocytosis) in KO acinar cells stimulated with CCK (500 pM) (Fig. 2A–E). In KO acinar cells, single and sequential compound exocytosis occurred at the apical portion, as in WT acinar cells. In fact, an increased [Ca^2+^]_i_, at levels of 15–20 µM, was evoked in the apical regions (Fig. 2D), and then an increase in SRB fluorescent intensity, indicating the primary exocytotic event, was induced at a certain latency (about 6 sec in Fig. 2C, E) after the calcium spike. Then, at the apical membrane, a sequential exocytosis was detected following the primary exocytotic event. In this process, an individual ZG fuses with an already-fused ZG, as represented by a second Ω-shaped profile (indicated by No. 2 in Fig. 2C) beginning to develop by fusion with the first Ω-shaped profile (indicated by No. 1 in Fig. 2C). Such sequential compound exocytosis involved up to five adjacent ZGs (data not shown). The morphology of these Ω-shaped profiles was similar between KO acinar cells and WT acinar cells, as also described in a previous report [18], while granule-granule fusion (i.e., the appearance of already-fused, not sequential, Ω-shaped profiles) was not evident in either KO or WT acinar cells.

To quantify the rate of progress of sequential compound exocytosis, we (a) defined the latency to the first exocytotic event at the apical plasma membrane (designated primary exocytosis) as the time (*L*_P_) from the onset of the increase in [Ca^2+^]_i_ to the formation of the first Ω-shaped profile (indicated by No. 1 in Fig. 2C), and (b) defined the latencies to the second, third, or fourth exocytotic events in the intracellular area located separately from the apical plasma membrane (designated secondary exocytosis) as the time (*L*_S_) between the corresponding sequential fusion reactions [in practice, the time between the formation of the first Ω-shaped profile (indicated by No.1 in Fig. 2C) and the appearance of the second Ω-shaped profile (No. 2 in Fig. 2C)]. Concerning *L*_P_, no significant difference was found between KO and WT acinar cells under any of the concentrations of CCK applied here (Fig. 2F). This suggests that the fusion competence of a ZG for primary exocytosis in the apical region was not affected by Noc2 deletion, because this latency (*L*_P_) predominantly represents the time required for exocytosis after a Ca^2+^ spike. Likewise, no significant difference in *Ls* was found between KO and WT acinar cells at any of the CCK concentrations, indicating that the fusion reaction of new ZGs to already-fused ZGs was not affected by Noc2 deletion.

Our finding that Noc2 deletion did not affect these latencies (viz., for primary exocytosis and secondary exocytosis) suggests that contrary to the prevailing notion, which regards Noc2 as a Rab effector, Noc2 is not essential for the progress of the membrane fusions involved in sequential compound exocytosis in the present cells.

### Frequency of zymogen-granule exocytosis induced by agonist stimulation and by an artificial Ca^2+^ increase

**Figure 3:**
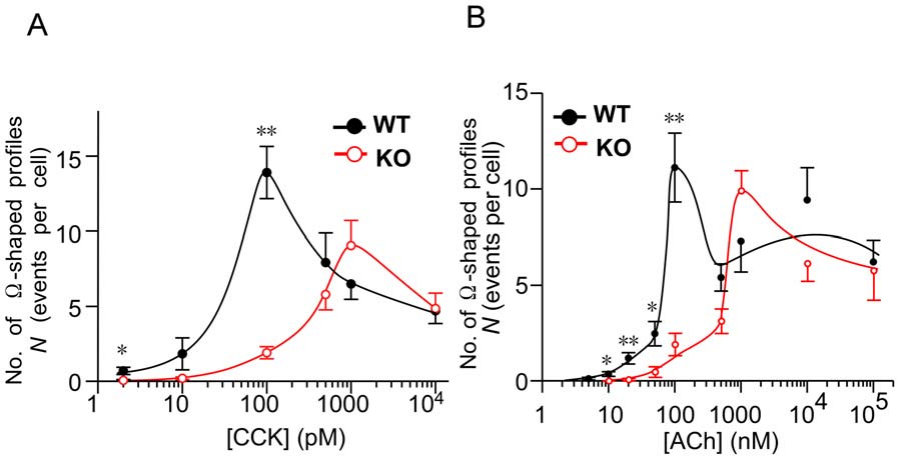
Number of exocytotic events induced in both strains by cholecystokinin and acetylcholine stimulation. *A, B*. Number of exocytotic events observed per cell per 10 minutes (*N*) in wild-type (WT; black filled symbols) and knockout (KO; red open symbols) acinar cells. Responses were induced by 10-min applications of various concentrations of cholecystokinin (CCK; A) or acetylcholine (ACh; B). Indexes * and ** indicate *p*<0.05 and *p*<0.01, respectively.

To explore further the observed impairment of ZG exocytosis, we examined the frequency of ZG exocytosis (Ω-shaped profiles) induced by CCK and by artificial Ca^2+^ increases. Surprisingly, at a physiological concentration (≤100 pM) of CCK stimulation, the average number (*N*) of such profiles in KO acinar cells was significantly less than that in WT acinar cells, although *N* was similar between the two strains at higher concentrations of CCK stimulation (Fig. 3A). To clarify whether defects in ZG exocytosis might depend on the type of agonist, we administered another natural agonist, acetylcholine (ACh). This agonist – not identical to, but similar to CCK – binds to its specific Gq-coupling receptor, and the two agents are thought to share the same downstream signaling pathway leading to IICR [2]. Under physiological concentrations of ACh (Fig. 3B), *N* was significantly less in KO acinar cells than in WT acinar cells, whereas it was similar between these cells under higher concentrations of ACh. Dose-dependency curves relating agonist concentration and *N* were similar between the two agonists. Thus, ZG exocytosis was found to be impaired in KO acinar cells at physiological concentrations of either of the agonists used here for stimulation.

**Figure 4:**
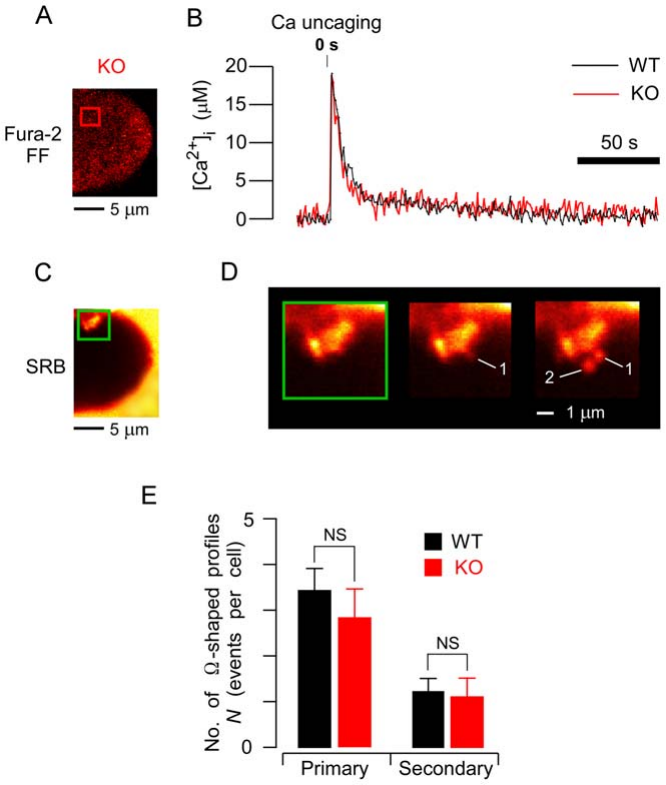
Number of exocytotic events in acinar cells of both strains following an artificial Ca^2+^ increase. *A, C.* Simultaneous cross-sectional fura-2 FF (A) and SRB images (C) of knockout (KO) acinar cells loaded with both fura-2 FF and NP-EGTA. *B*. Intracellular free Ca^2+^ concentration ([Ca^2+^]_i_) increases, induced by ultraviolet-light photolysis of caged calcium compound, in wild-type (WT; black line) and KO (red line; obtained from the area indicated by the red box in A) acinar cells. *D*. SRB-fluorescence images of a KO acinar cell showing sequential exocytotic events (Ω-shaped profiles 1, 2) 11 and 13 seconds after caged-calcium photolysis. Images are magnifications of the area enclosed by the green box in C, and were taken at the above times after calcium-ion uncaging. *E*. Numbers of primary and secondary exocytotic events observed per cell per 10 minutes (*N*) in WT (black bar; n = 20 cells) and KO (red bar; n = 18 cells) acinar cells. Responses were induced by caged-calcium photolysis. Data are means ± SE.

Next, we induced artificial [Ca^2+^]_i_ increases by flash photolysis in acinar cells loaded with the caged-Ca^2+^ compound NP-EGTA, and examined the frequency of ZG exocytosis. Such uncaging of this caged compound was able to generate a rapid and homogeneous increase in [Ca^2+^]_i_, mimicking the physiological increase that leads to sequential ZG exocytotic events in WT acinar cells. Thus, we could analyze the fusion step of ZG exocytosis in KO acinar cells, without prior activation of agonist-receptors and their downstream biomolecules [21]. Notably, such artificial increases in [Ca^2+^]_i_ were able to trigger frequent exocytotic events in KO acinar cells (Fig. 4A–D). When [Ca^2+^]_i_ increases within the physiological range (10–40 µM; 33.6±1.0 µM in KO acinar cells: n = 18; 32.2±1.6 µM in WT: n = 19; mean ± SE) were induced by uncaging, primary and secondary Ω-shaped profiles were induced (Fig. 4C, D). No significant differences in the number of primary or secondary exocytotic events were detected between KO and WT acinar cells (*p*>0.4, Fig. 4E). Thus, the exocytosis induced, without any activation of agonist-receptors, by an adequate artificial [Ca^2+^]_i_ increase was not impaired in KO (versus WT) acinar cells. This result supported our insight that Noc2 deletion does not affect the membrane-fusion steps involved in ZG exocytosis.

### Intracellular free Ca^2+^ concentration ([Ca^2+^]_i_) induced by agonist stimulation

**Figure 5:**
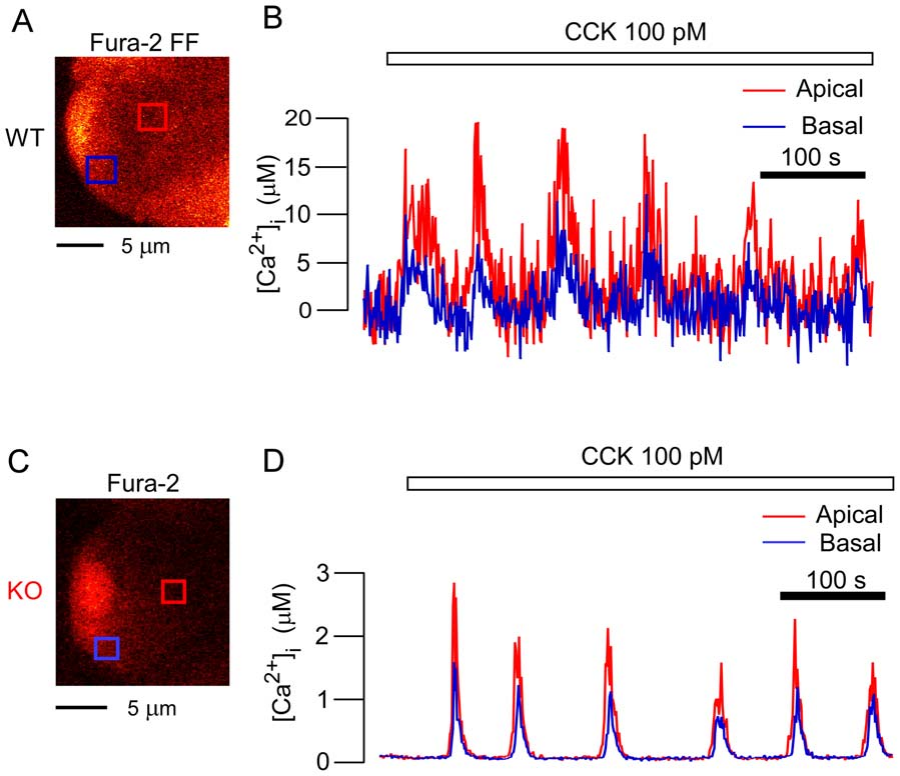
Ca^2+^ increases in acinar cells of both strains at a physiological concentration of cholecystokinin. *A, C.* Cross-sectional fluorescence images of wild-type (WT) acini loaded with fura-2 FF-AM (A) and of knockout (KO) acini loaded with the high-affinity Ca-indicator fura-2-AM (C). *B*. Time course of changes in [Ca^2+^]_i_ observed in apical (red box in A) and basal (blue box in A) regions of a WT acinar cell during application of 100 pM cholecystokinin (CCK). *D*. Time course of changes in [Ca^2+^]_i_ observed in apical (red box in C) and basal (blue box in C) regions of a KO acinar cell during application of the same concentration of CCK as in B. Note that ordinate scales differ markedly between panels B and D.

**Figure 6:**
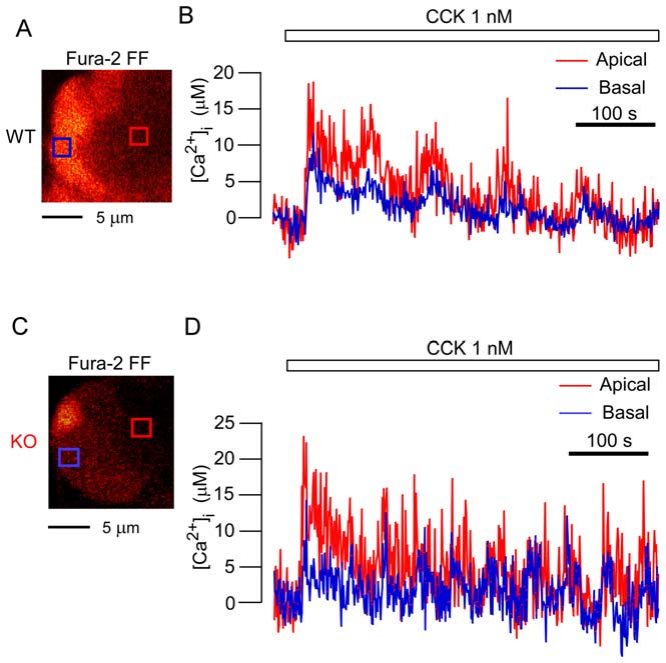
Ca^2+^ increases in acinar cells of both strains at a higher concentration of cholecystokinin. *A, C*. Cross-sectional fluorescence images of wild-type (WT; A) and knockout (KO; C) acini loaded with fura-2 FF-AM. *B.* Time course of changes in [Ca^2+^]_i_ in apical (red box in A) and basal (blue box in A) regions of a WT acinar cell during application of 1 nM cholecystokinin (CCK). *D.* Time course of changes in [Ca^2+^]_i_ in apical (red box in C) and basal (blue box in C) regions of a KO acinar cell during application of the same concentration of CCK as in B.

From the above results, some impairment(s) affecting a process other than the fusion step of ZG exocytosis must be supposed to be responsible for the decrease in ZG exocytosis shown by the present KO acinar cells. To assess the generation of calcium spikes, an upstream factor for ZG exocytosis, we measured the [Ca^2+^]_i_ levels from simultaneously acquired images of the two types of cells under CCK stimulation. In WT acinar cells under a physiological concentration of CCK (≤100 pM), an increase in [Ca^2+^]_i_ was detected that reached 15–20 µM (Fig. 5A, B). However, we could not detect such a significant increase in KO acinar cells using the low-affinity calcium indicator fura-2 FF, which was used for the experiments in WT acinar cells (data not shown). When the high-affinity calcium indicator fura-2 was employed, calcium spikes were detected in KO acinar cells, although the maximal level of micromolar [Ca^2+^]_i_ was below 3 µM (Fig. 5C, D). Thus, for us to be able to detect amplitude differences in the calcium signal between the acini of the two strains in response to physiological agonist concentrations, we needed to use two Ca^2+^ indicators that differed in their Ca^2+^-binding capacities. Under a higher concentration of CCK (1 nM), [Ca^2+^]_i_ reached 15–20 µM in the apical and basal regions of KO acinar cells (Fig. 6C, D) as well as in both of those regions in WT acinar cells (Fig. 6A, B). When a calcium spike occurred in KO acinar cells, it was detected first in the apical portion, from where it propagated to the basal portion, and calcium oscillations (repetitive calcium waves) also appeared (Fig. 5D and Fig. 6D). These were similar to those observed in WT acinar cells (Fig. 5B and Fig. 6B). These findings demonstrated that Noc2 deletion impaired the occurrence of calcium spikes only under a physiological concentration of CCK, and that Noc2 deletion did not alter the intracellular calcium-increasing and propagating mechanisms (site and mode of calcium spikes) under a higher concentration of CCK.

**Figure 7:**
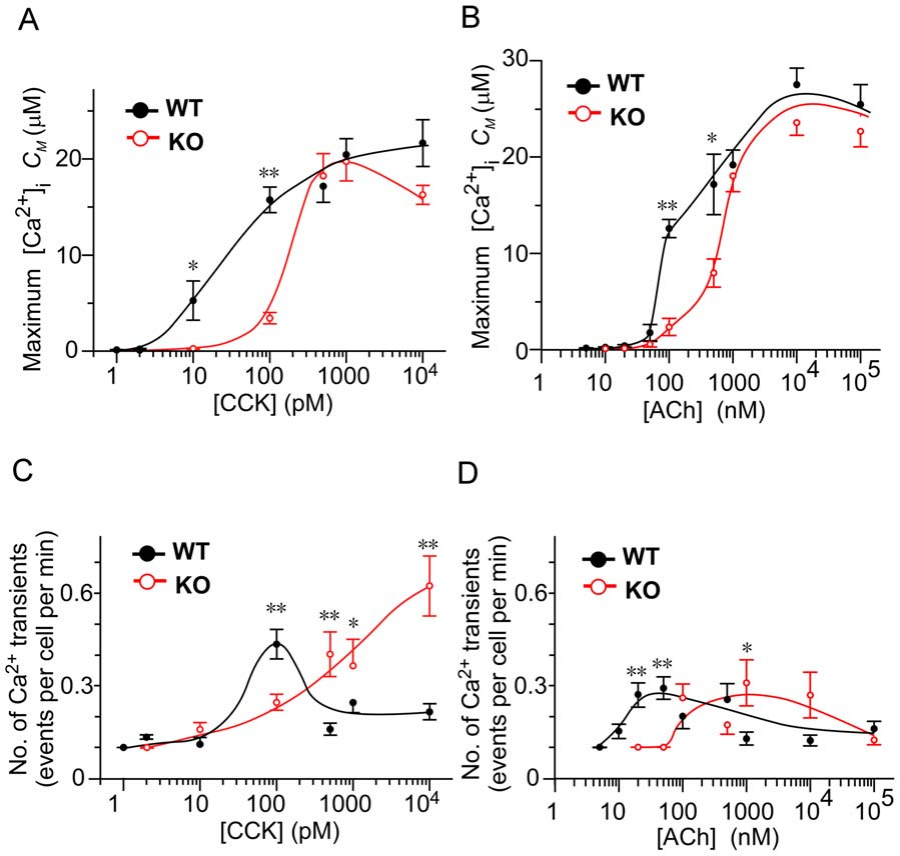
Ca^2+^ concentrations and transient numbers in acinar cells of both strains under agonist stimulation. *A, B*. Maximum values of intracellular free Ca^2+^ concentration ([Ca^2+^]_i_) [Ca^2+^]_i_ (*C_M_*) in wild-type (WT; black filled symbols) and knockout (KO; red open symbols) acinar cells. Responses were induced by 10-min applications of various concentrations of cholecystokinin (CCK; A) or acetylcholine (ACh; B). The values in (A) and (B) were obtained from the same samples as those in Fig. 3 (A) and Fig. 3 (B), respectively. Data are means ± SE of values obtained from the following numbers of cells: **in A**, 24 (1 pM), 23 (2 pM), 13 (10 pM), 21 (100 pM), 23 (500 pM), 18 (1 nM), or 21 (10 nM) different WT cells, and 17 (2 pM), 24 (10 pM), 12 (100 pM), 19 (500 pM), 14 (1 nM), or 21 (10 nM) different KO cells; **in B**, 26 (5 nM), 28 (10 nM), 27 (20 nM), 15 (50 nM), 17 (100 nM), 20 (500 nM), 17 (1 µM), 22 (10 µM), or 17 (100 µM) different WT cells, and 14 (10 nM), 19 (20 nM), 22 (50 nM), 28 (100 nM), 22 (500 nM), 31 (1 µM), 21 (10 µM), or 17 (100 µM) different KO cells. *C, D*. Average numbers of Ca^2+^ transients per minute in WT (black filled symbols) and KO (red open symbols) acinar cells. Responses were induced by 10-min applications of various concentrations of CCK (C) or ACh (D). Data are means ± SE of values obtained from the following numbers of cells: **in C**, 4 (1 pM), 6 (2 pM), 13 (10 pM), 21 (100 pM), 21 (500 pM), 18 (1 nM), or 18 (10 nM) different WT cells, and 1 (2 pM), 24 (10 pM), 12 (100 pM), 18 (500 pM), 14 (1 nM), or 14 (10 nM) different KO cells; **in D**, 4 (5 nM), 6 (10 nM), 10 (20 nM), 12 (50 nM), 9 (100 nM), 15 (500 nM), 7 (1 µM), 22 (10 µM), or 17 (100 µM) different WT cells, and 2 (20 nM), 6 (50 nM), 13 (100 nM), 8 (500 nM), 18 (1 µM), 18 (10 µM), or 17 (100 µM) different KO cells. Indexes * and ** indicate *p*<0.05 and *p*<0.01, respectively.

When we examined the relationship between the maximum [Ca^2+^]_i_ (*C*_M_) and the concentration of agonist, the average value of *C*_M_ was significantly lower in KO acinar cells than in WT acinar cells under physiological concentrations of CCK (≤100 pM) (Fig. 7A), while under higher concentrations of CCK (≥500 pM) the average was similar between KO and WT acinar cells. Under physiological concentrations of ACh, the curves relating agonist concentration and *C*_M_ were similar to those obtained for CCK (Fig. 7B). Under higher concentrations, although *C*_M_ tended to be lower in KO than in WT at 500 nM ACh, significance was not established at that concentration, and we concluded that values were similar between KO and WT acinar cells. These dose-dependency curves showed that in KO acinar cells, the value of *C*_M_ changed almost in parallel with the value of *N*. In the acini of each strain, the average number of Ca^2+^ transients per minute under either CCK (Fig. 7C) or ACh (Fig. 7D) stimulation showed evidence of a bell-shaped dependency on agonist concentration, as previously reported [18], although a down-slope after a peak was not confirmed in KO acini under CCK stimulation. These peaks in the number of Ca^2+^ transients occurred at higher agonist concentrations in KO acini than in WT acini (viz. WT 100 pM, KO 10 nM or more under CCK and WT 50 nM, KO 1 µM under ACh) even though these values did not differ significantly between KO and WT under CCK or under ACh. These results may suggest that KO acini have a calcium-generating system with a roughly normal integrity but a lower than normal sensitivity to agonist stimulation. If so, at physiological concentrations of CCK and ACh the observed defects in ZG exocytosis in KO acinar cells may be explained by their low [Ca^2+^]_i_ sensitivity in response to a given agonist. Moreover, Noc2 deletion reduced the response to two different agonists at physiological concentrations, suggesting that Noc2 deletion may interfere with a process shared by the receptors for those two agonists.

## Discussion

In the present study, we investigated the physiological roles played by Noc2 by means of two-photon excitation live-cell imaging. Our two-photon microscopy successfully demonstrated heavy accumulation of ZGs within KO acinar cells, and also that neither substantial ZG exocytosis nor [Ca^2+^]_i_ oscillations of ≥10 µM were evoked at physiological concentrations of natural agonists in such cells. These findings support the results obtained in a previous morphological study of KO pancreatic exocrine glands [13]. The accumulation of ZGs in KO cells may be supposed simply to be due to a weak response to stimuli for secretion, since the acinar cells of starved animals have been reported to accumulate more ZGs than those of feeding animals [26], [27]. As yet, the long-term effect of weak stimuli for ZG exocytosis on acinar cells is not known. Difficulty in evoking [Ca^2+^]_i_ increases during the cellular developmental stage may cause ZG accumulation within acinar cells. Furthermore, we cannot rule out the possibility of unknown mechanisms induced by Noc2 deletion causing ZGs to accumulate constantly within acinar cells.

It might seem a natural inference that the observed impairment of ZG exocytosis in KO acinar cells is caused by a defect in the membrane-fusion step of ZG exocytosis because Noc2 has been considered to operate as a Rab3D and/or Rab27B effector in the exocrine pancreas [23], [24], [28], [29]. However, in the present study, our two-photon microscopy yielded new findings that argue against such an interpretation. Although exocytosis was lost in KO pancreatic acinar cells at physiological, but not at higher agonist concentrations, our spatiotemporal analysis of the mode or latency of ZG exocytosis in the agonist-stimulation study and the normality of the ZG exocytosis in the photolysis experiment confirmed the integrity of the fusion step of ZG exocytosis. Simultaneous observation of [Ca^2+^]_i_ and ZG exocytosis revealed an impairment of the increase in [Ca^2+^]_i_, leading to a loss of ZG exocytosis in KO cells, suggesting that Noc2 plays a vital causal role in the increase in [Ca^2+^]_i_ that occurs upon physiological stimulation by agonists.

Contrary to previous assumptions, our results indicated that, in pancreatic acinar cells, Noc2 might not be involved directly, as a Rab effector, in the molecular machinery responsible for membrane fusion during sequential compound exocytosis of ZG, such as the lateral diffusion of SNARE proteins [18]. However, in the parotid exocrine gland, in which ZG exocytosis is triggered by a [Ca^2+^]_i_ increase, Noc2 complexed with Rab27 has been suggested to perform a positive mediating role in isoproterenol-stimulated ZG exocytosis [30]. Further investigation will be needed to establish whether such differences in the physiological functions of Noc2 among exocrine glands might be causally related to the expression level of Noc2. Concerning ZG exocytosis in the Noc2-deficient exocrine pancreas, we cannot exclude the possibility that other effectors, such as Slp1, might compensate for any loss of Rab function.

**Figure 8:**
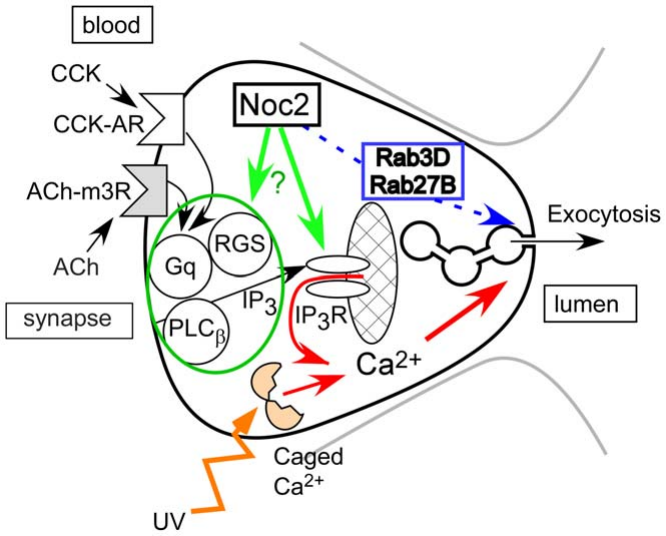
Proposed novel function of Noc2 in pancreatic acinar cells. Noc2 may not be essential as a Rab3D or Rab27B effector in ZG secretion (blue dotted line). Instead, it may be required for agonist-induced [Ca^2+^]_i_ release from the endoplasmic reticulum (cross-hatched ellipse) via inositol 1,4,5-trisphosphate receptor (IP_3_R) channels. An agonist-induced [Ca^2+^]_i_ increase may be induced either directly by Noc2 or via an indirect effect in which Noc2 interacts with other biomolecules. Abbreviations used are: CCK, cholecystokinin; CCK-AR, cholecystokinin type A receptor; ACh, acetylcholine; ACh-m3R, acetylcholine muscarinic type 3 receptor; Gq, heterotrimeric G protein Gq; IP_3_, inositol 1,4,5-trisphosphate; IP_3_R channel, inositol 1,4,5-trisphosphate receptor channel; PLCß, phospholipase Cß; RGS, regulator of G-protein signaling; UV, ultraviolet.

Our inference that Noc2 is involved in agonist-induced [Ca^2+^]_i_ increases raises intriguing questions since Noc2 does not itself have a calcium-binding site [11]. Structural changes due to massive ZG accumulation might conceivably influence agonist-induced [Ca^2+^]_i_ increases [31], although in the present study, at least, Ca^2+^ generation and the mode of propagation seemed not to be seriously impaired in KO acini. In general, agonist-induced [Ca^2+^]_i_ increases in pancreatic acinar cells are known to be mediated by the heterotrimeric G protein Gq, phospholipase Cß, and IP_3_, and different agonists can share an almost-identical downstream signaling pathway leading to IICR [32], [33]. Therefore, Noc2 deletion could conceivably operate in any of several ways. For example, it could impair a part of the agonist-induced Ca^2+^-release signaling process and/or the IICR for Ca^2+^-release from the ER at physiological concentrations (Fig. 8). Moreover, agonist-induced [Ca^2+^]_i_ increases could be influenced if the effectiveness of the above pathway were modulated by regulators for G protein signaling (RGS) and/or by changes in the open probabilities of the IP_3_-receptor channels on the ER [14], [34]–[36]. Furthermore, the expression level of membrane-receptors could be influenced by constitutive exocytosis, which involves another Noc2-binding partner, Rab8 [37]. Therefore, analysis of IP_3_/IP_3_-receptor function and of the distribution of cell-surface receptors in KO acini would be useful ways of clarifying the influence of Noc2 over the signal cascade. Future elucidation of the physiological function of Noc2 will require clarification of which biomolecule(s) might be feasible partner(s) in intact pancreatic acinar cells. Moreover, calcium dynamics in the pancreatic beta cells of Noc2 KO mice may be a profitable subject for investigation.

In conclusion, the present results suggest a novel function for Noc2: namely, that it is an essential factor in the mechanism responsible for agonist-induced Ca^2+^ increases in murine pancreatic acinar cells. Moreover, Noc2 may not contribute directly to membrane fusions during exocytosis in such cells. To clarify how Noc2 might contribute to agonist-induced Ca^2+^ release, binding partners and factors downstream of Noc2 will need to be identified in intact pancreatic acini *in vivo*. It will also be quite important to determine whether such a novel Noc2 function exists in other instances of regulated exocytosis in exocrine or endocrine glands, or indeed in the presynaptic terminals of neurons.
